# Cytohesins/ARNO: The Function in Colorectal Cancer Cells

**DOI:** 10.1371/journal.pone.0090997

**Published:** 2014-03-11

**Authors:** Tao Pan, Junfeng Sun, Jiyi Hu, Yiwang Hu, Jun Zhou, Zhigang Chen, Dong Xu, Wenhong Xu, Shu Zheng, Suzhan Zhang

**Affiliations:** 1 Department of Surgical Oncology, Second Affiliated Hospital, Zhejiang University School of Medicine, Hangzhou, China; 2 Cancer Institute (Key Laboratory of Cancer Prevention & Intervention, National Ministry of Education, Provincial Key Laboratory of Molecular Biology in Medical Sciences), Zhejiang University School of Medicine, Hangzhou, China; 3 Department of Gastrointestinal Surgery, Henan Cancer Hospital, Zhengzhou University, Zhengzhou, China; Seoul National University, Republic Of Korea

## Abstract

Epidermal growth factor (EGF) and insulin-like growth factor-I (IGF-I) are critical regulators of cell differentiation, survival, proliferation, and migration in cancers. This study found that ARNO (cytohesin-2), an activator of the EGF and IGF-I pathways, was more highly expressed in colorectal cancer tissue than in benign adjacent colorectal tissue. When ARNO-siRNA or the chemical inhibitor SecinH3 blocked ARNO, the downstream of the EGF and IGF-I pathways decreased in colorectal cell lines HT29 and HCT116. This blocking also weakened cell proliferation, invasion, and migration in vitro. Furthermore, EGF receptor (EGFR)-dependent colorectal tumor xenografts in nude mouse exerted anti-proliferative and growth suppression effects by injecting secineH3. These data suggested that inhibiting cytohesins or ARNO as cytoplasmic activators of EGFR and IGF-I in colorectal cancer resulted in anti-proliferation, reduced invasion, decreased migration, and suppressed growth in vivo and in vitro. Therefore, cytohesins or ARNO may be a potential therapy target for some colorectal cancer.

## Introduction

Colorectal cancer (CRC) remains to be the third most commonly diagnosed cancer in males and the second in females despite significant improvements in its prognosis ascribed to advances in diagnosis and therapy modalities. Over 1.2 million new cancer cases and 608 700 deaths are recorded annually [1]. The effective treatments of colorectal cancer are surgery, chemotherapy, and targeted therapy. Advances in conventional chemotherapy have extended life expectancy, but the effectiveness for many patients remains low, especially for those with metastasis. The search for more effective and less toxic therapies has given rise to a new generation of antitumor agents. The most common one is the targeted biological agent [2]. Epidermal growth factor (EGF) receptor (EGFR) and associated signal transduction pathways have emerged as important molecular therapeutic targets for colorectal cancer [3].

EGFR/ErbB1, along with Her2/ErbB2, Her3/ErbB3, and ErbB4, is a member of the ErbB family. EGFR/ErbB1 regulates the body's innate immune response [4] as well as cell differentiation, survival, proliferation, invasion, and migration. EGFR contains an extracellular ligand-binding domain, a single membrane-spanning region, and a cytoplasmic tyrosine kinase domain [5]; [6]. Ligands bind to the extracellular domain causing receptor dimerization, thereby inducing conformational change of intracellular phosphorylation components and enabling downstream signaling [7].

Nowadays, many targeted biological agents play important roles in the EGFR signaling pathway. Anti-EGFR monoclonal antibodies and EGFR tyrosine kinase inhibitors have been proven to efficiently inhibit the proliferation of cancers, especially colorectal and nasopharyngeal cancers [8]; [9]. Cetuximab and Panitumumab, antibodies against EGFR, are widely used to treat colorectal cancer. However, patients eventually develop resistance to these agents [10]. One common hypothesis of Cetuximab-resistance is EGFR or downstream molecular mutation within tumor cells, such as acquired EGFR ectodomain mutation S492R [10]. In addition, persistent EGFR blocking enhances pathways other than the EGFR pathway, such as the Her2, Her3, insulin-like growth factor (IGF)-I receptor (IGF –IR) signaling pathways [11]–[13].

IGF-I and IGF-II play central roles in cell growth, differentiation, survival, transformation, and metastasis. The biological effects of IGFs are mediated by IGF-IR, a receptor tyrosine kinase with homology to insulin receptor. Researchers recently found that the deregulation of the IGF system is a key contributor to the progression of multiple cancers, with IGF-IR activation increasing the tumorigenic potential of breast, prostate, lung, colon, as well as head and neck squamous cell carcinomas [14]; [15].

Cytohesins as activators of ErbB receptors have been reported by Bill et al. [16]. They showed that cytohesins enhance EGFR activation by directly interacting with the cytoplasmic domains of dimerized receptors and by facilitating the conformational rearrangements of these domains. Cytohesins over expression enhances EGFR signaling in human lung cancers, whereas the chemical inhibition or knockdown of cytohesins reduces EGFR activation. Similarly, our previous studies have shown that blocking cytohesins by SecinH3 or knocking down ARNO by ARNO-siRNA can reduce EGFR activation in the colorectal cancer cell lines HT29 [17]. EGF and IGFs are critical regulators of cell differentiation, survival, proliferation, and migration in cancers. They are also involved in the apoptosis, transformation, invasive growth, and distant metastasis of tumor cells [15]. Cytohesins have been suggested as a new effective target for reducing invasion, metastasis, and Cetuximab or Panitumumab-resistant cells in colorectal cancer patients. The possibility that cytohesins can be new targets for drug-resistant or advance-stage cancer patients have been explored. Accordingly, we examined cytohesins or ARNO as a new anti-colorectal cancer agent in this study.

## Materials and Methods

### Antibodies and reagents

The cell culture media 1640 and McCoy S 5A were purchased from Gibco (Gibco, USA). The rabbit or mouse monoclonal anti-human antibodies used were ARNO (Abcam, ab56510), pEGFR (Py1068, Epitomics, 1138-1), pERK1/2 (T202/Y204, Bioworld, BS5016), EGFR (Cell Signaling, 3197), GAPDH (Bioworld, AP0063), IGF-IR (Abcam, ab39675), pIGF-IR (Abcam, ab39398), pIRS (Abcam, ab52167), pAKT (Abcam, ab106693), pIRS1 (Abcam, ab66153), pShc (Abcam, ab155170), and Ki-67(Cell Signaling, 9027). Other reagents and equipment used were as follows: SecinH3 (Merck-565725/sc-203260), siRNA oligo (Genephama), MTT (sigma, m5655), DMSO (sigma, D5879), human EGF (Peprotech, AF-100-15), human IGF-1 (Peprotech, AF-100-11), FBS (Gibco, USA), and 0.25% trypsin (Sigma), immunohistochemical kit(Zhongshan,China).

### Cell lines and cultivation

The human colorectal cancer cell lines HT29 and HCT116, which were identified without any mutation in KRAS and BRAF, were obtained from the Key Laboratory of Cancer Prevention and Intervention, Cancer Institute, Second Affiliated Hospital, School of Medicine, Zhejiang University, China. The cell cultures used were as follows: HT29 cell line by 1640 (with 10% FBS + 1% streptomycin/penicillin) and HCT116 cell line by McCoy 's 5A (with 10% FBS + 1% streptomycin/penicillin). All cell lines were cultured in a 37°C 5% CO_2_ incubator and passaged with 0.25% trypsin (Sigma) in 0.2 M phosphate-buffered saline (PBS).

### Tumor Samples

Before the human tumor samples research, the patients' informed consent must had been obtained. We had submitted a statement from our hospital ethics committee and received the approval of the research. All tumor samples stem from the Biobank at the Key Laboratory of Cancer Prevention and Intervention, Cancer Institute, Zhejiang University, China. All tumors were clinically and pathologically identified as being the primary and only neoplastic lesion and classified according to World Health Organization (WHO) Classification of Tumors of the Digestive System (2010).

### Immunostaining

For immunostaining, we used rabbit monoclonal antibodies raised against ARNO (Abcam, ab56510), mouse antibodies against pEGFR (Py1068, Epitomics, 1138-1), rabbit polyclonal antibodies against pIGF-IR (Abcam, ab39398) and rabbit monoclonal antibodies against Ki-67(Cell Signaling, 9027) as primary antibodies. Before application, all antibodies were diluted (Primary antibodies diluted 1∶100, all other secondary antibodies diluted 1∶200) in PBS (150 mM NaCl, 10 mM Na_2_HPO_4_, 10 mM NaH_2_PO_4_, pH 7.4). Immunohistochemistry was performed according to the manufacturer's instructions. Staining intensities were individually evaluated by three independent observers using a four-tier scoring system as described [18].

### MTT

HT29 or HCT116 cells were seeded onto 96-well plates at a density of 3000 cells/well. Cells were cultured with 1% FBS and reagents (50 ng/ml EGF or 25 ng/ml IGF-1, SecinH3 with different concentration (0 µM, 10 µM, 20 µM, 40 µM)) for 24, 48, and 72 h at 37°C and 5% CO_2_. Then 5 mg/ml MTT was added to each well and incubated for 4 h. Then 200 µl of DMSO was added to resolute MTT substrate, and absorbance was measured at 570 nm using a Spectra MAX micro plate reader (Bio-Rad, USA)

### Western blot analysis

Cells were collected and extracted with a eukaryotic cell lysis buffer according to the manufacturer's instructions. Proteins were separated by 12% SDS-PAGE and blotted onto a nitrocellulose membrane with a wet transfer device (Bio-Rad, Hercules, CA, USA). Blotted membranes were blocked with 10% skimmed milk in PBS Tween-20 for 1 h. After washing the membranes three times with Tris-buffered saline Tween-20 (TBST), they were incubated with primary antibody diluted 1∶1000 at room temperature for 1 h and then incubated in HRP-labeled secondary antibody diluted 1∶10 000 at room temperature for 1 h. After rinsing the membranes, visualization was conducted with an enhanced chemiluminescence Western blot analysis system (Amersham Biosciences, Little Chalfont, UK), and cells were exposed to X-ray film (Kodak). GAPDH protein was used as an inner control. The bolts are detected and analysis by software Alpha Imager EP (Version: 3.2.2.0).

### Cell migration assays

Cell migration assays were performed using 24-well Tran swell plates (8 µm pore size; Costar). About 1×10^4^ cells (HT29 or HCT116) were loaded into the upper chambers. The lower chambers were filled with medium (1640 plus 1% FBS) in the absence (DMSO 0.2%) or presence of SecinH3 (10, 20, or 40 µM). The Tran swell plates were then incubated in a 37°C, 5% CO2 incubator for 48 h. After cleaning the cells from the upper side of polycarbonate membrane and hematoxylin–eosin staining, the polycarbonate membrane was cut and placed on a microscope slide, cover slipped, and examined under the microscope. The total migrated cell number and percentage were then counted.

### Xenograft tumor models

All animal procedures were conducted in accordance with the Zhejiang University Laws for Animal Protection and approved by the Zhejiang University animal protection committee. Tumors were generated by subcutaneous injections of 5×10^6^ HT29 cells into nu/nu athymic male mice according to Ullrich et al. [19]. After establishing tumors (about 6 mm in diameter), fourteen mice were randomized into two groups. Mice in the SecinH3 group were treated with daily intraperitoneal injections of SecinH3 (100 µl, 2.5 mM; in 75% glucose solution (5%)/25% DMSO). Mice in the control group were treated with the same volume of 75% glucose solution and 25% DMSO until the 14th day. During treatment, we measured the biggest tumor diameter every 2 days by ultrasound and then sacrificed the mice to collect the tumors. We then performed immunohistochemical staining for Ki-67 to detect the proliferation inhibition of SecinH3 in the xenograft tumor models. The total Ki-67 positive cell number and percentage were then counted.

### Statistics

Results are given as the mean ± standard error of the mean (SEM). The statistical software SPSS16.0 was used for statistical analysis. Paired comparisons were performed by Student's *t*-test. Pearson correlation analysis was conducted, with *p*<0.05 (marked “*”) and *p*<0.01 (marked “**”) considered significantly different or significantly correlated.

## Results

### ARNO over expression was correlated with EGFR and IGF-IR levels in human colorectal cancer tissue

**Figure 1:**
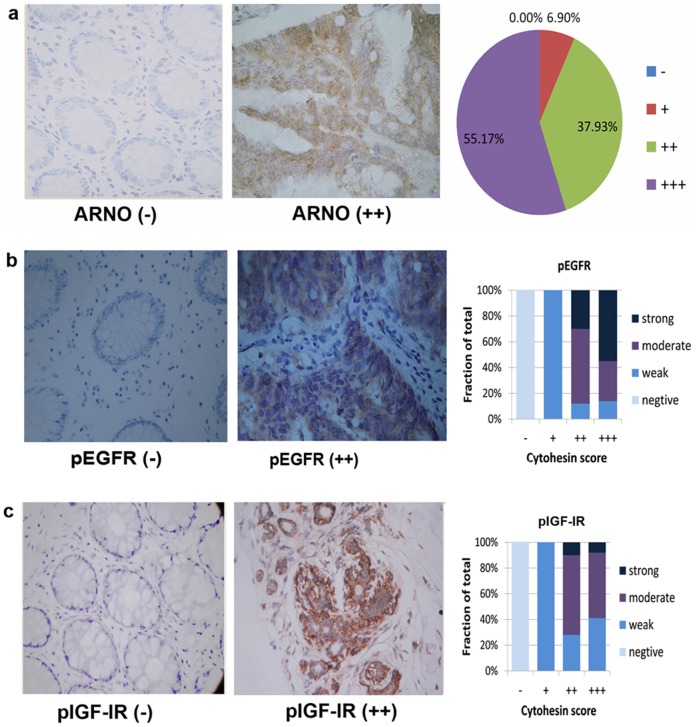
High expression levels of ARNO were correlated with increased EGFR and IGF-IR signaling in human colorectal adenocarcinomas. Patients' colorectal cancers or benign adjacent tissue consecutive sections were stained for ARNO (a), pEGFR (b), and pIGF-IR (c). Representative images of normal colorectal tissue (left column) and moderate (right column) ARNO expression are shown (original magnification ×100). The diagram in (a) shows the fraction and frequencies of tumors with background (−), weak (+), moderate (++), or strong (+++) staining for ARNO. The diagrams in (b) and (c) depict the correlation of the phosphorylation levels of respective proteins with the ARNO score (*p* = 0.012 for pEGFR, *p* = 0.031 for pIGF-IR, *n* = 36). It reveals that ARNO over expression was correlated with enhanced EGFR and IGF-IR.

EGFR and IGF-IR signaling play critical roles in many types of cancer [16] and our group found that ARNO and other cytohesins enhance EGFR activation in the colorectal cancer cells [17]. Thus, we wondered whether ARNO was over expressed in patients' cancer tissues and the over expression was correlated with EGFR and IGF-IR signaling. Therefore, we used immunohistochemistry to investigate primary human colorectal adenocarcinomas with an antibody detecting ARNO, pEGFR (pY1068), and pIGF-IR (pY1185). Results showed that normal colorectal tissue or benign adjacent colorectal tissue had only background (30/36) or weak staining (6/36). Moreover, all carcinomas showed ARNO positive staining, 93.1% of which were moderate or strong. We found a highly significant (*r* = 0.712, *p* = 0.012 for pEGFR; *r* = 0.684, *p* = 0.031 for pIGF-IR, *n* = 36) correlation between the expression level of ARNO and pEGFR or pIGF-IR (Fig.1). Our previous studies on the role of ARNO in colorectal cancer tissues have revealed a high correlation with pEGFR and pIGF-IR, suggesting that ARNO possibly enhances the activation and signaling of EGFR and IGF-IR.

### Chemical inhibition of cytohesins and knockdown of ARNO reduced cell signaling

**Figure 2.Cytohesins/ARNO:**
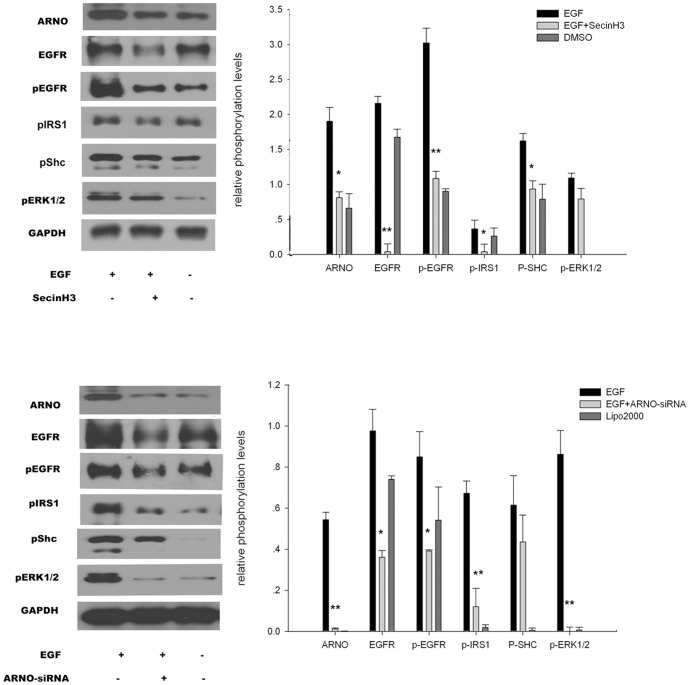
ARNO enhanced the activation of EGFR. (a and b) SecinH3 and ARNO-siRNA reduced EGFR receptor signaling. Western blot analysis of HT29 cells treated with SecinH3 (a) or ARNO-siRNA (b) and stimulated with EGF is shown. Phosphorylation of the indicated proteins was determined by immunodetection using phosphospecific antibodies. Glyceraldehydes phosphate dehydrogenase (GAPDH) served as a loading control. The diagrams show relative phosphorylation levels after normalization for GAPDH. The untreated ligand-stimulated cells were set as 3 (*n* = 3). Data is represented as the mean ± SEM. * *p*<0.05, **p<0.01.

To detect the function of cytohesins or ARNO in the EGF pathway of colorectal cancer cells, SecinH3 and ARNO-siRNA (selected by the primary experiments) inhibit cytohesins/ARNO in HT29 cells [17]. In the assay, HT29 cells were cultured in 35 mm glass-bottom dishes marked as group A, B, or C. All cells were cultured with 1% FBS culture medium. SecinH3 (20 µM or a mixture of 100 pmol of ARNO-siRNA in 5 µl of Lipofectamine 2000) was added to dishes from group B when cells had spread to cover 70% of the dishes for 10 h. Simultaneously, 0.2% DMSO (or 5 µl of Lipofectamine 2000) was added to dishes from groups A and C as a control, and then 50 ng/ml EGF was added to dishes from groups A and B for 5 min. Western blot analysis was used to test the expression of EGF pathway-associated molecules, including ARNO, EGFR, pEGFR, pIRS1, pShc, and pERK1/2. Results indicated that when SecinH3 blocked cytohesins or ARNO inhibited by ARNO-siRNA, ARNO expression was reduced in HT29 cells. Additionally, the phosphorylated molecules of the EGF pathway including pEGFR, pShc, and pERK1/2 were downregulated in HT29 cells (Fig. 2).

**Figure 3:**
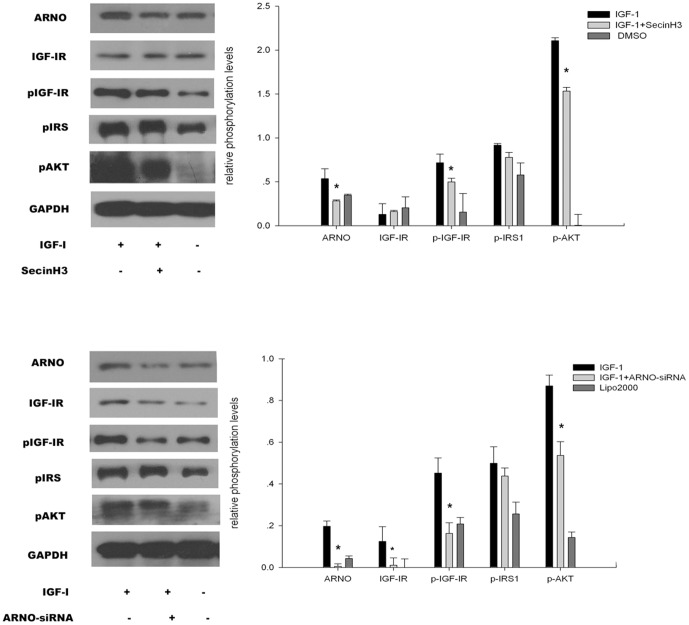
Cytohesins/ARNO enhanced the activation of IGF-IR. (a and b) SecinH3 and ARNO-siRNA reduced IGF-IR receptor signaling. Western blot analysis of HCT116 cells treated with SecinH3 (a) or ARNO-siRNA (b) and stimulated with IGF-1 is shown. Phosphorylation of the indicated proteins was determined by immunodetection using phosphospecific antibodies. Glyceraldehydes phosphate dehydrogenase (GAPDH) served as a loading control. The diagrams show relative phosphorylation levels after normalization for GAPDH. The untreated ligand-stimulated cells were set as 3 (*n* = 3). Data are represented as the mean ± SEM. * *p*<0.05, **p<0.01.

To detect the function of cytohesins or ARNO in the IGF pathway, SecinH3 and ARNO-siRNA [17] in HCT116 cells inhibited cytohesins/ARNO. In the assay, HCT116 cells were cultured in 35 mm glass-bottom dishes marked as group A, B, or C. All cells were cultured with 1% FBS culture medium. SecinH3 (20 µM or a mixture of 100 pmol of ARNO-siRNA in 5 µl of Lipofectamine 2000) was added to dishes from group B when cells had spread to cover 70% of the dishes for 10 h. Simultaneously, 0.2% DMSO (or 5 µl Lipofectamine 2000) was added to dishes from groups A and C as a control, and then 25 ng/ml IGF-1 was added to dishes from groups A and B for 5 min. Western blot analysis was used to test the expression of IGF pathway-associated molecules including ARNO, IGF-IR, pIGF-IR, pIRS, and pAKT. Results indicated that when cytohesins were blocked by SecinH3 or inhibited by ARNO-siRNA, ARNO expression was reduced in HCT116 cells. Additionally, phosphorylated molecules of the IGF pathway including pIGF-IR and pAKT were downregulated in HCT116 cells (Fig. 3).

### Blocking cytohesins reduced the proliferation and migration of colorectal cells

**Figure 4:**
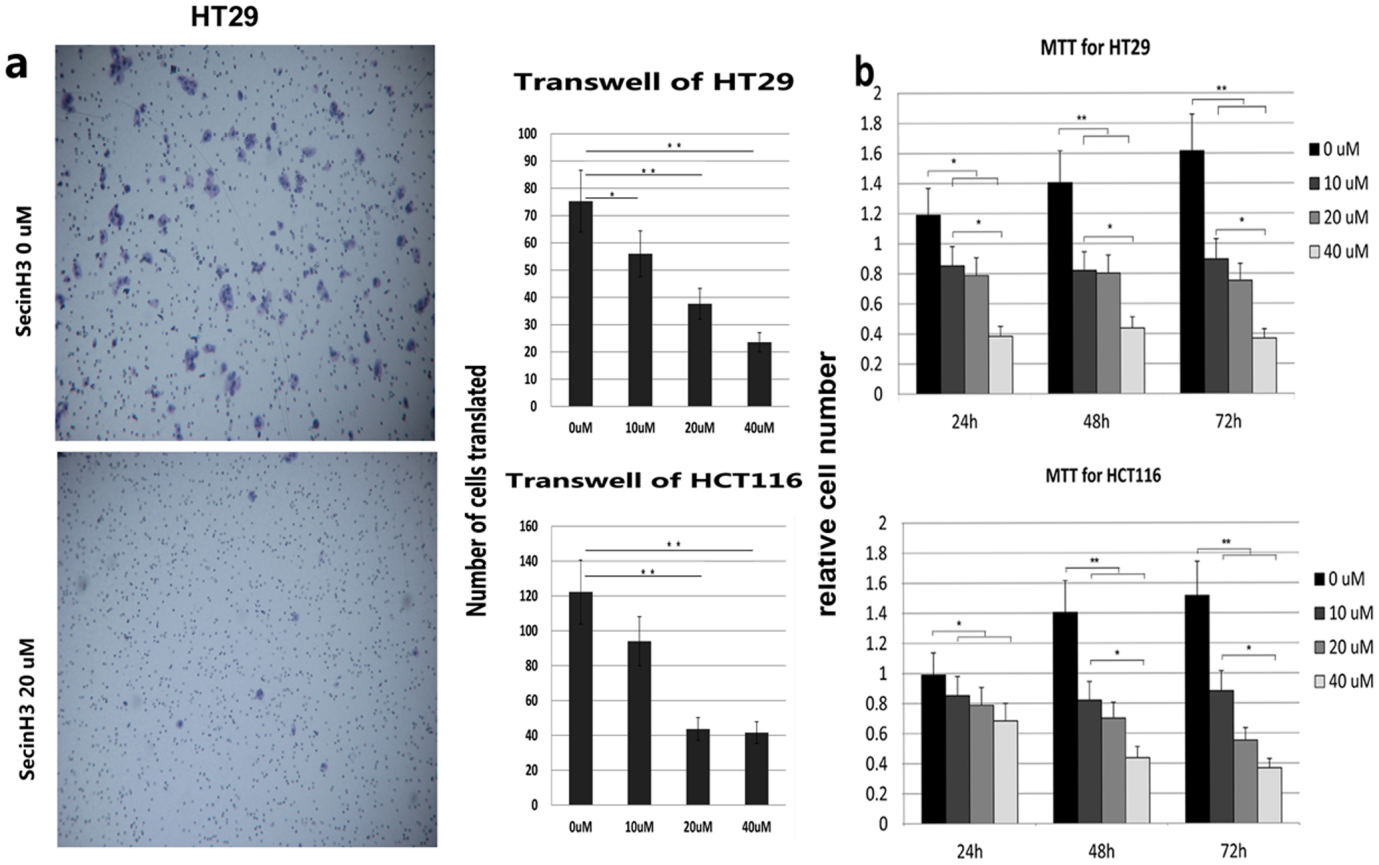
SecinH3 reduced the proliferation and migration of HT29 and HCT116. Left column of diagram (a) are representative images of Tran swell assay of HT29 and HCT116 cells treated with SecinH3 in 0, 10, 20, and 40 µM respectively. The left pictures are the result of HT29 cells without SecinH3 (DMSO 0.2% for 48h) and with SecinH3 (20 µM for 48 h) (original magnification ×100). It seems that SecinH3 can inhibit the migration of HT29 and HCT116 cells. The results are correlation with the concentration of SecinH3 and the time. The diagrams (b) show relative cell number of HT29 and HCT116 determined by MTT assay in the presence of SecinH3 in 0, 10, 20, and 40 µM for 24, 48, and 72 h. It seems that SecinH3 can inhibit the infiltration of HT29 and HCT116 cells. The results are also correlation with the concentration of SecinH3 and the time. Data is represented as the mean ± SEM. * p<0.05, **p<0.01 (*n* = 5).

Immunohistochemistry of patients' cancer tissues revealed that ARNO over expression was correlated with enhanced EGFR and IGF-IR. This finding prompted us to think about the possibility of reduced proliferation or migration of EGFR/IGF-sensitive cells when ARNO is blocked. To select EGFR/IGF-sensitive cell lines, we performed MTT assay by culturing the colorectal cancer cell lines HT29, SW620, SW480, HCT116, and LOVO in 1% FBS with EGF or IGF. We found that HT29 was the EGFR-dependent cell line and HCT116 was IGF sensitive (data not shown). Both cell lines were identified as wild type for KRAS and BRAF (data not shown). To detect the relationship of ARNO with cell proliferation and migration in colorectal cancer, we added sesinH3 to the culture medium and found it can reduce the proliferation (MTT assay with SecinH3 in 0(DMSO), 10, 20, and 40 µM for 24, 48, and 72 h) (Fig. 4b), and migration (Tran swell with SecinH3 in 0(DMSO), 10, 20 and 40 µM for 48 h) of HT29 and HCT116 cells (Fig. 4a). It shows that SecinH3 can inhibit the infiltration and migration of HT29 and HCT116 cells. And the effects are proportional to the concentration of SecinH3 and the time of operation.

### SecinH3 reduced the growth of colorectal HT29 tumor xenografts

**Figure 5:**
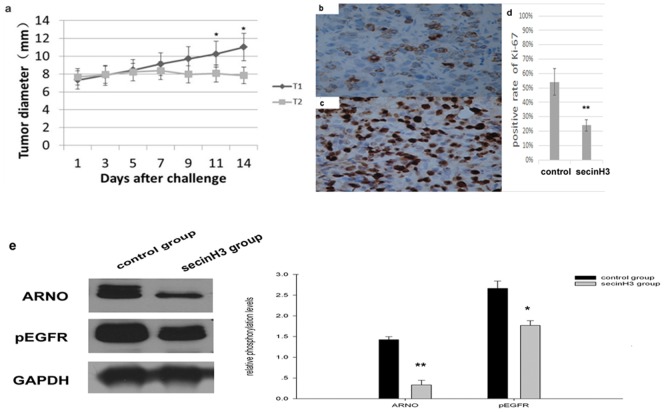
SecinH3 reduced the growth of colorectal HT29 tumor xenografts. The diameter of xenograft tumor established in mice was measured by ultrasound every 2 days during treatment (diagram a). T_1_ was the control group. T_2_ was the SecinH3 group. The tumors of the SecinH3 group were inhibited growth obviously, after daily intraperitoneal injections of SecinH3 for more than a week. There was significant difference between the two groups in the 11^th^, 14^th^ days after treatment. Diagram (b) and(c) represent immunohistochemical straining results for Ki-67 of tumor from mice after treated with (b) or without SecinH3(c) for 14 days, (original magnification ×200). It seams that SecinH3 decreases the expression of Ki-67 in HT29 xenografts. Diagram (d) depicts the positive expression rate of Ki-67 in the tumors of mice bearing HT29 xenografts in the 14^th^ day after treatment with or without SecinH3. Diagram (e) depicts the positive expression of ARNO, pEGFR in the tumors of mice bearing HT29 xenografts after treatment with or without SecinH3 for 2 weeks. The ARNO and pEGFR expression of the two groups have significant difference. The diagrams show relative phosphorylation levels after normalization for GAPDH. Data are represented as the mean ± SEM. *p<0.05, * *p<0.01, n = 7.

The strong expression of ARNO in colorectal cancer tissue and its significant correlation with pEGFR and pIGF-IR prompted us to wonder whether blocking cytohesins in vivo can inhibit the proliferation of tumor cells. To investigate this hypothesis, HT29 cells that have the higher expression of EGFR than other colorectal cancer cells [17], was selected to do the xenograft mouse model. So we subcutaneously injected HT29 cells into nude mice to generate tumor xenografts. When the tumor xenograft size reached 6–7 mm in mice that had been injected the HT29 cells for almost a week, the mice began to treat with or without SecinH3. The tumors of the SecinH3 group were inhibited growth obviously after treatment with SecinH3 for more than a week. The two groups had obvious differences until the 11th days after the treatment (Fig. 5a). Immunohistochemical staining of the cell proliferation marker Ki-67 in resected tumors confirmed the reduced cell proliferation. The total Ki-67 positive cell number and percentage were then counted. We found the highly significant different expression level between of the mice treated with or without SecinH3 after the treatment of 14 days (p = 0.0083, n = 7) (Fig.5b, c, d). Western blot analysis was used to test the expression of EGF pathway-associated molecules in tumors mice treated for 2 weeks, including ARNO, pEGFR. Results indicate that when SecinH3 blocked cytohesins, ARNO and pEGFR expression were reduced in HT29 xenografts. (Fig.5e).

## Discussion

EGF and IGF are critical regulators of the biological characteristics of cells, especially in cancers [20]. Our previous study has shown that ARNO is the most important cytohesin and is over expressed in colorectal cancer cell lines as an activator that plays a crucial role in EGFR pathway signaling [17].

To verify the hypothesis that ARNO is related to colorectal cancer by activating EGF and IGF, we performed immunohistochemistry of resected human colorectal adenocarcinomas stained by ARNO, pEGFR, and pIGF-IR. Compared with normal colorectal tissue or benign adjacent colorectal tissue, ARNO, pEGFR, and pIGF-IR were over expressed in adenocarcinomas. We also found a highly significant correlation between the expression levels of ARNO and pEGFR or pIGF-IR.

Additionally, we used siRNA and SecinH3-inhibited ARNO/cytohesins in colorectal cancer cell lines to explore the activity of ARNO and its association with the EGFR and IGF-IR signaling system. We then detected the downstream of EGFR and IGF-IR in association with colorectal cancer incidence rates. When we inhibited cytohesins or ARNO, the downstream molecules of EGF pathway were mirrored in the reduced activation of pEGFR, pIRS1, pShc, and pERK1/2. Evidence suggested that blocking cytohesins or ARNO could reduce the EGF pathway system. At all times, we detected IGF pathway downstream including IGF-IR, pIGF-IR, pIRS, and pAKT. These molecules were down regulated when ARNO was inhibited chemically or by siRNA. These down regulations indicated that signal amplification and transduction pathways were efficiently inhibited [21]; [22]. Thus cytohesins or ARNO was strongly correlated with EGF and IGF pathway activation in colorectal cancer [23].

In a cellular context, we used the human colorectal cancer cell lines HT29 and HCT116 identified without any mutation in KRAS and BRAF. When ARNO-siRNA or SecinH3 blocked ARNO or cytohesins, the proliferation of colorectal cancer cells were reduced in MTT. Furthermore, the proliferation reduction was positively correlated with the SecinH3 concentration. For the invasion and migration assay, Tran swell assay was performed. The pores in the Tran swell membranes were blocked with a gel (matrigel) composed of extracellular matrix to mimic the typical matrices that tumor cells encounter during the invasion process in vitro. By placing the colorectal cells on the upper side of the gel to be attracted by a higher serum concentration on the other side of the well, invasion was determined by counting those cells that had traversed the cell-permeable membrane having invaded and migrated toward the higher concentration serum. In this assay, we found that inhibiting cytohesins by SecinH3 decreased the invasion and migration of colorectal cancer cells. Researchers have recently reported similar reductions in lung and prostate cancer [6]; [19]; [24]. This reduction can contribute to the down regulation of EGF and IGF-I pathway signal amplification and transduction. In the in vivo study, we injected SecinH3 daily after the HT29 tumor xenografts were generated in nude mice. We found that the growth of tumor was obviously inhibited after 9 days of SecinH3 injection. After 14 days of treatment, tumor proliferation in mice was also inhibited.

In a recent research, it is find that ARNO is highly expressed in colorectal cancer, and the expression is correlated with the EGFR and IGF-IR pathways. ARNO inhibition can reduce the signaling and conduction of these pathways, as well as decrease the proliferation, invasion, and migration of colorectal cancer cells in vivo and vitro. Ludovini [25] reported that if both IGF-IR and EGFR are highly co expressed in resected non-small-cell lung cancer, patients might achieve shorter disease-free survival. Choi et al. [26] indicated that combined inhibition of IGF-IR signaling enhances the growth inhibitory and apoptosis-inducing effects of EGFR pathway inhibitor. However, cancer cells always have many signal channel ways to reproduce, and EGFR and IGFR are only two of these ways. Although ARNO may be a new therapy target of some colorectal cancer cells, the higher concentration of ARNO in cancer cells than in normal cells may be due to other reasons such as proliferation and immunity. The mechanism of migration may also be related to integrin β [27]. All these hypotheses need to be researched in the future.
