# Correction: Cytohesins/ARNO: The Function in Colorectal Cancer Cells

**DOI:** 10.1371/journal.pone.0146204

**Published:** 2015-12-28

**Authors:** Tao Pan, Junfeng Sun, Jiyi Hu, Yiwang Hu, Jun Zhou, Zhigang Chen, Dong Xu, Wenhong Xu, Shu Zheng, Suzhan Zhang

In the Funding section, the grant number from the funder Zhejiang Provincial Natural Science Foundation of China is listed incorrectly. The correct grant number is: LQ13H160015.
